# Current Approach to the Diagnosis and Treatment of Femoral-Popliteal Arterial Disease. A Systematic Review

**DOI:** 10.2174/157340309789317823

**Published:** 2009-11

**Authors:** Christos Kasapis, Hitinder S Gurm

**Affiliations:** Division of Cardiovascular Medicine, University of Michigan, Ann Arbor, MI, USA

**Keywords:** Peripheral arterial disease, superficial femoral artery, popliteal artery, diagnosis, management.

## Abstract

Peripheral arterial disease (PAD) is a common manifestation of atherosclerosis affecting 5 million adults in the United States, with an age-adjusted prevalence of 4% to 15% and increasing up to 30% with age and the presence of cardiovascular risk factors. In this article we focus on lower extremity PAD and specifically on the superficial femoral and proximal popliteal artery (SFPA), which are the most common anatomic locations of lower extremity atherosclerosis. We summarize current evidence and perform a systematic review on the diagnostic evaluation as well as the medical, endovascular and surgical management of SFPA disease.

## INTRODUCTION

Peripheral arterial disease (PAD) is a common manifestation of atherosclerosis and is defined as any pathologic process causing obstruction to blood flow in the arteries, exclusive of the coronary and cerebral vascular beds [1]. PAD affects a large segment of the adult population, with an age-adjusted prevalence of 4% to 15%, affecting more than 5 million adults in the United States [2, 3] and increasing up to 30% with age (Fig. (**1**)) and the presence of cardiovascular risk factors [4]. Less than 20% of patients with PAD have typical symptoms of intermittent claudication –i.e., leg-muscle discomfort on exertion that is relieved by rest–, or critical limb ischemia – i.e., “rest pain”, ulceration or gangrene– [4, 5], whereas another third have atypical exertional leg symptoms [6].

Age and gender-adjusted risk factors associated with the development of PAD are similar to the traditional risk factors for atherosclerosis, including cigarette smoking, diabetes, hyperlipidemia, hypertension, hyperhomocystinemia and chronic renal insufficiency (Fig. (**2**)) [1, 2, 7]. Smoking and diabetes mellitus are the strongest risk factors and are associated with more aggressive disease progression. The relative risk of intermittent claudication is 3.7 in current smokers and 3.0 in ex-smokers [8], whereas the presence of diabetes increases the risk of PAD by 2- to 4-fold with 5- to 10-times higher need for major amputation in diabetics compared to non-diabetics [2, 9].

Studies of the natural history of PAD indicate that the risk of ischemic limb events for non-diabetic patients is relatively low, with less than 2% requiring major or minor amputation [1, 7, 10]. However, cardiovascular disease and manifestations of atherosclerosis in other vascular beds are the major determinants of prognosis in these patients. There is approximately a 2- to 4-fold increase in coronary and cerebrovascular disease in patients with PAD, with an annual rate of 5-7% of cardiovascular events, i.e. myocardial infarction, stroke, or death from cardiovascular causes [1, 5, 7, 11]. Notably, the risk of cardiovascular morbidity and mortality is equally high in patients with PAD, regardless of the presence of symptoms [5, 12]. Therefore, management of the disease should be directed not only at improving symptoms of claudication and lower limb ischemia, but more importantly at reducing the overall cardiovascular risk.

In this article we focus on lower extremity PAD and specifically on the superficial femoral and proximal popliteal artery (SFPA), which are the most common anatomic locations of lower extremity atherosclerosis. We summarize current evidence on the diagnostic evaluation as well as the medical, endovascular and surgical management of SFPA disease.

## DIAGNOSTIC EVALUATION

The initial evaluation should include a careful history and physical examination with shoes and socks removed to assess for signs of acute or chronic peripheral ischemia, with attention to peripheral pulses, hair loss, skin color and trophic skin changes. The abdomen should be examined for evidence of an aortic aneurysm and blood pressure should be measured in both arms. Auscultation for bruits at the neck and over the clavicles, abdomen and femoral pulses should also be performed. Upon clinical suspicion for PAD, referral to the vascular laboratory is the initial step for noninvasive diagnostic assessment of the location and severity of the arterial disease (Fig. (**3**)). These noninvasive tests can also be repeated over time to follow disease progression and response to medical treatment or revascularization.

### Ankle-Brachial, Toe-Brachial Indices and Segmental Pressure Measurements

The ankle-brachial index (ABI) is the single best initial screening test to perform in a patient with suspected PAD. The ABI is the ratio of the highest systolic ankle pressure to the highest arm pressure (in mmHg), obtained with a hand-held Doppler instrument and a blood pressure cuff [1, 7]. A ratio of <0.90 is considered abnormal, with mild obstruction defined as a ratio of 0.71-0.90, moderate as 0.41-0.70 and severe when the ABI ratio is less than 0.40. Both arm pressures must be recorded, since even in normal individuals, there is a minimal (less than 12 mmHg) inter-arm systolic pressure gradient which may be amplified in patients with atherosclerotic subclavian or axillary arterial disease. Subsequently, the higher arm blood pressure is used for the ABI ratio calculation. In addition, pulse wave reflection causes the ankle pressures to be 10-15 mmHg higher than the brachial systolic pressure, which gives a normal ABI of greater than 1.00. The overall accuracy of the ABI to establish the diagnosis of lower extremity PAD has been validated with a sensitivity of 79-95% and a specificity of 96-100%, based on different studies [13, 14]. Overall, it is considered to have low inter-observer variability with a reproducibility of approximately 0.10. Apart from establishing the PAD diagnosis, the ABI also has prognostic value in identifying patients at risk for subsequent critical leg ischemia as well as cardiovascular morbidity and mortality and can also be used to assess disease progression after vascular surgery [1]. Furthermore, it is a quick and cost-effective way to screen targeted patients at risk for lower extremity PAD, including individuals 50 years or younger with history of diabetes and one other risk factor; those 50 to 69 years with history of smoking or diabetes; those 70 years and older; and those with abnormal pulse examination or known atherosclerotic disease in other vascular beds [1, 4, 7, 9].

ABI measurement in the vascular lab is used in conjunction with segmental pressure measurements with plethysmographic cuffs placed sequentially along the limb at various locations to accurately determine the location of individual artery stenosis. A systolic pressure gradient greater than 20 mmHg between adjacent segments is considered in most laboratories to be indicative of a physiologically important focal stenosis.

The major limitation of the ABI and the segmental pressure analysis is that they may be inaccurate in individuals with noncompressible arteries due to medial calcification, such as patients with long-standing diabetes, elderly patients and those with end-stage renal disease on dialysis. Such non-compressible arteries should be suspected when the ABI is greater than 1.3 or when the lower extremity systolic pressure is more than 20% higher than the brachial systolic pressure. In such cases, accurate diagnostic information can be obtained by calculating the toe-brachial index and values less than 0.7 are considered diagnostic for lower extremity PAD [1, 7, 15]. The test is performed by placement of a small cuff on the proximal portion of the great or second toe and measurement of the systolic perfusion pressure with the use of a plethysmographic device.

### Pulse-Volume Recordings and Continuous-Wave Doppler Ultrasound

Pulse volume recordings (PVR) are based on the concept that arterial inflow into the lower extremities is pulsatile leading to measurable changes in the lower-limb volume with each cardiac cycle. These volumetric changes can be documented and graphed sequentially along the limb with the use of a plethysmographic technique. The magnitude of the pulse upstroke and pulse volume (amplitude) correlates with the blood flow and a sequential decrease signifies the presence of a flow-limiting lesion in the more proximal arterial segment.

Continuous-wave Doppler ultrasound is used in conjunction with the PVR to obtain segmental velocity waveforms and systolic blood pressure measurements along the upper or lower extremities. A commonly used term is the peak-to-peak pulsatility index, defined as the peak systolic velocity minus the minimum or most reversed diastolic velocity, divided by the mean blood flow velocity. A decrease in the pulsatility index between the adjacent proximal and the distal arterial segments indicates the presence of physiologic significant stenosis. One important limitation is the pulse normalization distal to some arterial stenoses, within a short distance from the lesion [16]. The analysis of the morphology of the Doppler waveform can also provide useful localizing information, i.e. the combination of low-resistance Doppler waveform and low pulsatility index within an arterial segment would indicate an occlusion proximal to that segment. One notable exception is the presence of superficial femoral artery disease, where, in the absence of aortoiliac disease, there is a low resistance waveform and low pulsatility index in the common femoral artery resulting in a false positive study suggesting aortoiliac disease [17].

Both PVR and Doppler waveform techniques can provide accurate information even in patients with noncompressible vessels and are used to establish the initial diagnosis, assess the location and severity of lower extremity PAD as well as to follow the progression after a revascularization procedure [1, 7].

### Treadmill Exercise Testing with and without ABI

Exercise testing can be useful in establishing the diagnosis of PAD when there is a high index of suspicion and the resting ABI measurements are normal, i.e. in patients with isolated iliac stenosis. A decrease in ABI of 15%-20% with exercise would be diagnostic of PAD [1, 7]. Most laboratories use motorized treadmills, however, alternative forms of exercise such as active pedal-plantar flexion, climbing stairs, walking in the hallway or the 6-minute walk test can also be employed. Moreover, exercise can help to objectively assess the degree of functional limitations posed by PAD, differentiate claudication from pseudoclaudication in patients with exertional leg symptoms, monitor functional improvement in response to claudication interventions and guide individualized exercise prescriptions in patients with PAD [1].

### Noninvasive Imaging Techniques

Duplex ultrasound provides both vascular imaging and flow velocity information and has been shown to be an accurate method to diagnose the location and severity of lower extremity PAD with both sensitivity and specificity greater than 90% [18]. Accuracy is diminished for the iliac arteries in the presence of bowel gas or tortuosity as well as if there is dense calcification or multiple stenoses. Quantitative criteria to diagnose stenoses are based on peak systolic velocity and peak systolic velocity ratios within or beyond the stenosis compared with the adjacent proximal arterial segment, the presence or absence of turbulence and preservation of pulsatility. A peak systolic velocity ratio greater than 2 indicates stenosis greater than 50% [19-21]. Apart from the initial diagnosis of the location and degree of stenosis, duplex ultrasound is recommended for routine surveillance after surgical revascularization with a venous conduit at 1, 3, 6 and 12 months and then yearly thereafter [1, 7]. On the contrary, duplex ultrasound surveillance of synthetic grafts as well as after angioplasty procedures is of uncertain value [1].

Recent advances in noninvasive angiography, including computed tomographic angiography (CTA) and magnetic resonance angiography (MRA), enable excellent noninvasive definition of the vascular anatomy to diagnose the location and degree of stenosis in PAD. New multi-detector CTA technology allows fast imaging of the entire lower extremity and abdomen in one breath hold at sub-millimeter voxel resolution and it can also provide 3-dimensional reconstruction images that can be rotated in space to view any oblique projection. Both sensitivity and specificity for detection of stenosis greater than 50% have been greater than 90% in different studies [22-27].

The potential advantages of CTA over conventional angiography include better evaluation of eccentric stenoses and visualization of all collateral vessels as well as surrounding tissues. On the other hand, potential disadvantages include lower spatial resolution compared to catheter angiography and venous opacification or asymmetrical opacification of some vessels that can obscure imaging quality of the arteries. Compared to MRA, CTA has the potential advantages that patients with pacemakers and defibrillators can be imaged safely; it has higher resolution; fewer artifacts with metallic clips, stents or prostheses; lower cost; and it can provide images of calcification in the vessel wall [28, 29]. Conversely, disadvantages of CTA include the use of iodinated contrast and higher risk of nephrotoxicity as well as exposure to ionizing radiation.

In a similar manner, MRA of the extremities can be used to diagnose PAD of the lower extremities with high accuracy similar to that of intraoperative catheter angiography [30]. A meta-analysis comparing MRA with catheter angiography demonstrated that sensitivity and specificity of MRA for detection of stenoses greater than 50% were both in the range of 90-100% [31]. Some studies suggest that MRA is superior to catheter angiography in detection of outflow vessels suitable for distal bypass in patients with chronic limb ischemia [32, 33]. MRA may be used for preoperative planning, to define a road map for revascularization procedures and has been used anecdotally for follow-up assessment after surgical or endovascular revascularization [1]. However, MRA has also inherent limitations in that it tends to overestimate the degree of stenosis because of turbulence; it cannot scan patients with pacemakers or defibrillators; metal stents or clips can obscure vascular flow; and use of gadolinium was recently associated with rare occasions of renal toxicity and nephrogenic systemic fibrosis in patients with elevated creatinine [34].

### Contrast Angiography

Contrast angiography provides detailed information about the arterial anatomy and is recommended as the “gold standard” method for evaluation of patients with lower extremity PAD, especially when revascularization is contemplated. Technical improvements with digital subtraction techniques that enhance image quality as well as use of supportive modalities, such as intravascular ultrasound, angioscopy, optical coherence tomography and adjunctive hemodynamic measurements, such as pressure gradients, established catheter angiography as the universally accepted method for guiding percutaneous peripheral interventional procedures [1, 7]. Angiography carries the inherent risks associated with any invasive procedure, including those related to vascular access (e.g. bleeding, infection, atheroembolism, dissection, vessel disruption or perforation, hematoma, pseudoaneurysm, arteriovenous fistula) with rates of major complications ranging from 1.9 to 2.9%, which is further reduced with operator experience [35]. Anaphylactoid reactions occur in fewer than 3% of cases with the risk of severe reactions of approximately 0.1% [36]. The risk of contrast induced nephropathy is less than 2% in the general population, but increases considerably in high-risk patients with severe baseline renal dysfunction, diabetes, low cardiac output, dehydration, advanced age, multiple myeloma or those receiving other nephrotoxic drugs [37, 38]. Optimal prevention of contrast induced nephropathy requires vigorous hydration with either 0.9% isotonic normal saline or isotonic sodium bicarbonate solution, use of low-osmolar contrast agents, minimizing the overall amount of contrast and pretreatment with N-acetylcysteine; various other modalities are investigated with unclear effectiveness [37].

## MANAGEMENT

**Fig. (4):**
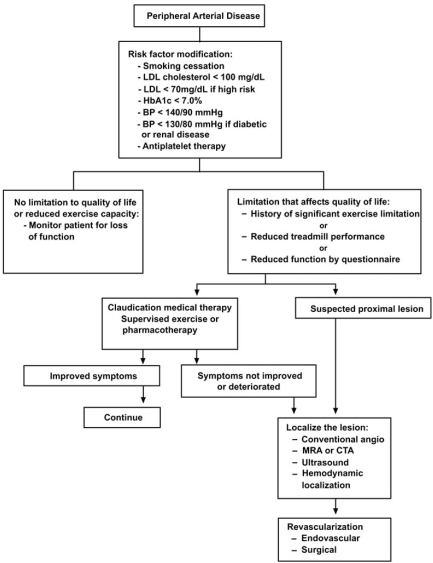
Overall treatment strategy for peripheral arterial disease. BP – blood pressure; HbA1c – hemoglobin A1c; LDL – low density lipoprotein; MRA – magnetic resonance angiography; CTA – computed tomographic angiography. Reproduced with permission from Hiatt WR. N Engl J Med 2001; 344: 1608-21.

**Fig. (5):**
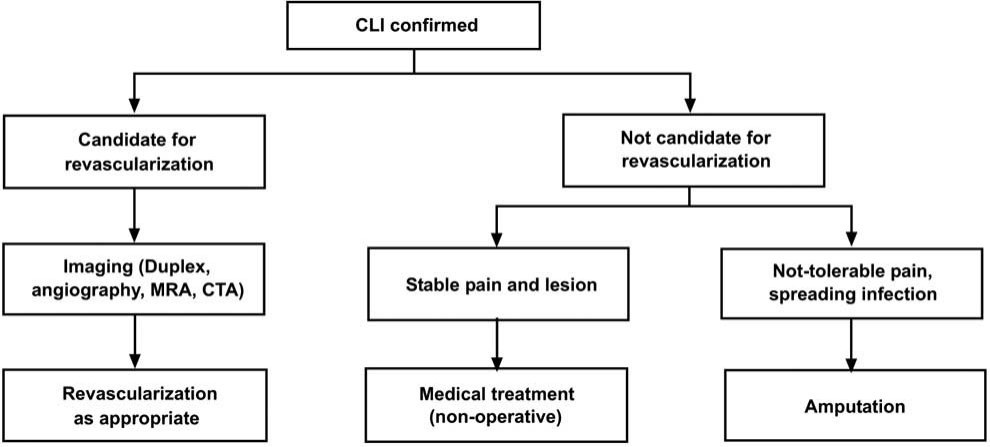
Algorithm for treatment of the patient with critical limb ischemia. Contraindications are: patients not fit for revascularization; revascularization not technically possible; benefit cannot be expected (i.e. widespread ulceration-gangrene). CLI – critical limb ischemia; MRA – magnetic resonance angiography; CTA – computed tomographic angiography. Reproduced with permission from [7].

The mainstays of treatment of lower extremity PAD and specifically of SFPA disease include risk factor modification, an exercise program, pharmacologic treatment and, if warranted for symptomatic relief, endovascular or surgical revascularization (Figs. **4** and **5**). In general, revascularization is reserved for patients with significant disability or tissue loss, failure of medical therapies, lack of significant comorbid conditions, vascular anatomy suitable for planned revascularization and a favorable risk/benefit ratio [1, 7].

### Risk Factor Modification and Cardiovascular Risk Reduction

Since cardiovascular events are the major cause of death in patients with PAD [1, 5, 7, 11, 12], modification of traditional atherosclerotic risk factors is of paramount importance in managing these patients. Particular emphasis should be given on smoking cessation and aggressive glycemic control in diabetic patients, since both represent the most dominant risk factors for PAD.

Smoking cessation among patients with symptomatic PAD does not improve walking capacity but may reduce the severity of claudication and the risk of developing critical limb ischemia [39]. Although there are no prospective randomized trials to examine the effects of smoking cessation on cardiovascular events in patients with lower extremity PAD, given its strong association with both PAD and cardiovascular disease, it is a class I recommendation in current guidelines that comprehensive smoking cessation interventions should be offered to all patients with lower extremity PAD, including behavior modification therapies, nicotine replacement therapy or bupropion [1, 7].

Within the same concept, aggressive glycemic control in diabetics with a target of hemoglobin A1C less than 7% is endorsed by both the American College of Cardiology/American Heart Association (ACC/AHA) and the Trans-Atlantic Inter-Society Consensus (TASC II) for the management of PAD as well as the American Diabetes Association [1, 7, 9]. Although studies of both type 1 and type 2 diabetes traditionally have shown that aggressive blood glucose lowering can prevent microvascular complications, this has not been shown for PAD, primarily because these studies were neither designed nor powered to examine PAD endpoints [40, 41]. However, more recently, an extended follow-up of the Diabetes Control and Complications Trial [41] demonstrated that intensive glycemic control in type 1 diabetics can provide long-term beneficial effects on the risk of cardiovascular disease [42]. Furthermore, it has been estimated that each 1% increase in glycosylated hemoglobin level is associated with a 32% and 28% increased risk of incident PAD in type 1 and 2 diabetics, respectively [43].

In addition to smoking cessation and aggressive management of diabetes, treatment of dyslipidemia reduces the risk of cardiovascular events in patients with atherosclerosis. There is plenty of evidence from large randomized trials and meta-analyses [44-47] demonstrating the beneficial effects of statin treatment in reducing the incidence of claudication and improving pain-free walking time, apart from the well-recognized reduction in overall cardiovascular morbidity and mortality. Moreover, a revision of the National Cholesterol Education Program Adult Treatment Panel III guidelines classifies individuals with PAD as either “high risk” or “very high risk” depending on the presence of coexisting risk factors and recommends a target LDL cholesterol <100 or <70 mg/dL for these patients, respectively [48]. These targets are also corroborated by the recent ACC/AHA and TASC II guidelines for the management of PAD [1, 7].

Similarly, hypertensive patients with PAD should be treated accordingly to meet current guidelines targeting blood pressure less than 140/90 mmHg; or less than 130/80 mmHg for patients with diabetes or chronic kidney disease [1, 7, 49]. Contrary to the prior belief, b-adrenergic blockers do not adversely affect walking capacity nor worsen intermittent claudication in patients with mild and moderate PAD and may be particularly beneficial in patients with diminished left ventricular function [50]. Moreover, angiotensin-converting enzyme inhibitors and specifically ramipril have been shown to reduce the risk of myocardial infarction, stroke or vascular death by 25% in patients with PAD [51]. Ramipril has also been associated with improvement in walking distance in stable claudicants in a single study and should be considered in all patients with symptomatic PAD who do not have any contraindications to this class of drugs [52]. 

### Exercise and Rehabilitation in Patients with PAD

Exercise significantly improves walking time and overall walking ability in patients with intermittent claudication and should be considered as a primary efficacious treatment in PAD [1, 7, 53, 54]. Notably, exercise may be more effective than antiplatelet therapy in improving maximal walking time and has equivalent efficacy to surgery [54]. Furthermore, physical activity both prevents and helps treat many established atherosclerotic risk factors, including elevated blood pressure, insulin resistance and glucose intolerance, elevated triglyceride concentrations and low high-density lipoprotein [55]. There is also a well-recognized role of habitual physical activity in reducing inflammation, which is implicated in the pathogenesis of atherosclerosis [56]. The optimal exercise training program for patients with intermittent claudication involves supervised walking to near-maximal pain 3 times a week at least for 30 minutes sessions for a period of 6 months. A meta-analysis of eight randomized trials demonstrated a greater symptomatic benefit with a supervised, as opposed to non-supervised, exercise program, involving walking on a treadmill three times a week for 12 to 24 weeks, with the initial workload set to elicit symptoms within 3 to 5 minutes of walking [57]. The mechanism by which exercise improves symptoms of claudication is unclear, but it appears to be unrelated to improvement of the ABI or growth of collateral vessels [58].

### Antiplatelet Therapies

The Antithrombotic Trialists’ Collaboration meta-analysis of 135,640 patients at high risk of occlusive arterial disease demonstrated that antiplatelet therapy with aspirin (75 mg to 325 mg daily) reduces the risk of myocardial infarction, stroke and cardiovascular death by 25% in patients with PAD [59]. In the same meta-analysis, different doses of aspirin were compared suggesting that 75 to 150 mg daily doses are at least as effective as higher doses with lower risk of gastrointestinal and bleeding complications. Aspirin has not been shown to improve claudication, but it delays the progression of the disease, reduces the need for intervention and reduces graft failure in patients who have undergone surgery [59, 60].

The Clopidogrel vs. Aspirin in Patients at Risk of Ischemic Events (CAPRIE) trial demonstrated that clopidogrel 75 mg daily compared with aspirin 325 mg daily reduced the risk of myocardial infarction, stroke or cardiovascular death by 8.7% with similar bleeding complications in high risk patients, with the greatest benefit in the subgroup of patients with PAD [61]. On the basis of this single comparative trial and considering the higher cost, current guidelines recommend clopidogrel as an effective alternative antiplatelet therapy to aspirin [1, 7].

Dual antiplatelet therapy with a combination of aspirin and clopidogrel in high risk patients with PAD was recently evaluated and it was not found significantly more effective than aspirin alone in reducing the rate of myocardial infarction, stroke, or death from cardiovascular causes, whereas there was a trend for higher severe bleeding events in the dual antiplatelet therapy group [62]. Similarly, ticlopidine, an alternative thienopyridine to clopidogrel, is not supported by the current guidelines, because of higher risk of significant hematologic side effects, including thrombocytopenia, thrombotic thrombocytopenic purpura and potentially fatal neutropenia [1, 7]. Likewise, there is no benefit of heparin, low-molecular weight heparin or oral anticoagulants in patients with PAD, whereas there is an increased risk of major bleeding, especially with oral anticoagulants [63]. Furthermore, the combination of oral anticoagulant and antiplatelet therapy in patients with PAD was demonstrated to be not more effective than antiplatelet therapy alone in preventing major cardiovascular complications, while it was associated with an increase in life-threatening bleeding [64].

Use of antiplatelet therapy after an endovascular or surgical revascularization procedure has been shown to improve outcomes, although the evidence is not conclusive [65-68]. However, based on the overall reduction of the cardiovascular risk, current guidelines recommend lifelong antiplatelet medications (aspirin or clopidogrel) after a revascularization procedure to promote patency. Furthermore, standard therapy during endovascular interventions with heparinization is recommended to achieve an activated clotting time of 200-250 msec [7]. The use of alternative antithrombotic agents during peripheral vascular interventions, such as low molecular weight heparin or adjunctive abciximab for complex lesions, has been investigated with equivocal results.

### Pharmacologic Treatment for Claudication

Cilostazol is a phosphodiesterase type III inhibitor with vasodilator and mild antiplatelet properties. A meta-analysis of eight randomized controlled trials demonstrated that treatment with cilostazol for 12 to 24 weeks significantly increased maximal and pain-free walking distance by 50% and 67%, respectively, and improved quality of life measures in patients with PAD [69]. The recommended dose is 100 mg twice daily in patients with intermittent claudication and is contraindicated in patients with heart failure, because of an associated increase in cardiac mortality in this subgroup seen with other phosphodiesterase inhibitors, such as milrinone [1].

Pentoxifylline, a methylxanthine derivative, has also been approved for use in patients with intermittent claudication, as alternative to cilostazol, at a dose of 400 mg three times a day. In a trial comparing cilostazol, pentoxifylline and placebo, pentoxifylline was inferior to cilostazol and no better than placebo for relief from claudication [70]. However, two meta-analyses of randomized controlled trials demonstrated that pentoxifylline causes a marginal but statistically significant increase in pain-free and maximal walking distance [39, 71].

Naftidrofuryl, a 5-hydroxytryptamine type 2 antagonist, has been available in European countries for treatment of intermittent claudication [7]. In a meta-analysis of 5 studies, involving 888 patients, naftidrofuryl at a dose of 600 mg daily increased pain-free distance by 26% compared to placebo [72], whereas in a different meta-analysis there was a more marginal but significant increase in maximal and pain-free walking distances [39].

Other investigated medical treatments, such as garlic, testosterone, levocarnitine, propionyl-L-carnitine, L-arginine, oral vasodilator prostaglandins, vitamin E, ginkgo biloba and chelation therapy have been evaluated in clinical trials and have not shown to be effective or are marginally or less effective than the currently established treatments [1, 7]. 

### Angiogenic Growth Factors

Angiogenic growth factors, such as vascular endothelial growth factor (VEGF), recombinant fibroblast growth factor-2 (rFGF-2) and hypoxia-inducible factor-1, have generated considerable enthusiasm as potential treatments of PAD that stimulate the development of new vessels. Intra-arterial infusion of rFGF-2 resulted in a significant increase in peak walking time in 90 days; however, repeat infusion in 30 days was no better [73]. Although there were no adverse events in this study, one prior placebo-control study of intravenous administration of basic fibroblast growth factor was terminated prematurely because of the development of significant proteinuria in 25% of the subjects [74]. Conversely, initial studies have not been positive for intramuscular administration of VEGF [75]. Newer applications involve gene transfer strategies of angiogenic proteins with viral or non-viral vectors [76]. Plasmid adjuncts, such as poloxamers, have also been used to enhance gene expression [77]. However, more studies will be needed to address the overall efficacy and safety as well as the mode and frequency of administration of angiogenic growth factors in the treatment of PAD.

### Endovascular Treatments and Revascularization

Over the past decade there has been a remarkable advancement in the endovascular treatment of lower extremity PAD with the introduction of new interventional techniques and devices. According to ACC/AHA guidelines, endovascular treatment of SFPA disease is indicated for individuals with significant disability due to intermittent claudication or critical limb ischemia when clinical features suggest a reasonable likelihood of symptomatic improvement with endovascular intervention, there has been an inadequate response to exercise or pharmacological therapy and when there is a favorable risk-benefit ratio [1]. Apart from the clinical and angiographic criteria for selection of patients for endovascular treatment, for stenoses of 50-75% diameter by angiography, intravascular translesional pressure gradients have been recommended to determine whether these lesions are hemodynamically significant and to predict patient improvement after revascularization. Although there is no consensus on the diagnostic translesional pressure gradient criteria, the most widely accepted criteria utilize a mean gradient of 10 mmHg before or after vasodilators; or a mean gradient of 5 mmHg and peak systolic gradient of 10, 15 or 20 mmHg; or 15% peak systolic pressure gradient after administration of a vasodilator [78]. The TASC II consensus emphasizes more anatomic criteria and recommends endovascular revascularization for type A lesions and surgery for type D lesions, whereas endovascular treatment is preferred for type B lesions and surgery for good-risk patients with type C lesions (Fig. (**6**)) [7]. There is a wide variety of established and evolving endovascular techniques to treat PAD including percutaneous transluminal angioplasty (PTA) with balloon dilation, stents, endografts, atherectomy, laser, cutting balloons, drug-coated balloon angioplasty, cryoplasty, percutaneous thrombectomy and brachytherapy.

#### Percutaneous Transluminal Angioplasty

From all the above techniques, PTA is the preferred recommended initial endovascular treatment for SFPA disease, with provisional stenting or use of other adjunctive techniques as salvage therapy for a suboptimal or failed result from balloon dilation, i.e. persistent translesional pressure gradient, residual diameter stenosis greater than 50%, or flow-limiting dissection [1, 7].

The technical and clinical success rate of PTA with provisional stenting for SFPA disease exceeds 93% [79]. Outcomes after PTA have improved over time, with primary patency rates at 1 year ranging from 45% to 84.2% and at 2 years from 25% to 77.2% [80]. In the STAR registry the technical success rate for PTA was 95%, with primary patency rates of 87% at 1 year, 80% at 2 years, 69% at 3 years and 55% at 4 and 5 years. Stratified to TASC categories, the 36-month patency rate was 87% for TASC A lesions, 69% for TASC B lesions and 67% for TASC C lesions, indicating that TASC C lesions may be treated with PTA with results similar to TASC B lesions [81]. Predictors of failure of endovascular treatment include critical limb ischemia, distal location, occlusion, poor crural runoff vessels, increasing length of lesions, multiple and diffuse lesions, diabetes, smoking and renal insufficiency [81-83].

Several randomized controlled trials compared the outcomes of PTA versus surgery in patients with SFPA disease and demonstrated similar outcomes [84-86]. More recently, the BASIL trial (Bypass versus Angioplasty in Severe Ischemia of the Leg), a large multicenter study of 452 patients with severe limb ischemia randomized to surgery or angioplasty, showed similar outcomes in terms of amputation-free survival with surgery being more expensive than angioplasty [87]. Similarly, a cost-effectiveness analysis compared PTA and bypass surgery with exercise therapy for treatment of claudication and demonstrated that the cost-effectiveness for PTA was $38,000 per quality-adjusted life year compared to $311,000 per quality-adjusted life year for bypass surgery [88]. In regards to cost-effectiveness, PTA is preferable to surgery as long as the expected 5-year patency rate for the treated vessel exceeds 30% [89]. In addition, PTA is preferred over surgery, when possible, in patients younger than 50 years old, because they have a higher risk of graft failure after surgical therapy than do older patients [1].

#### Stents

The role of primary stent placement in SFPA disease remains controversial. Endovascular stenting avoids the problems of early elastic recoil, residual stenosis and flow-limiting dissection after balloon angioplasty and can thus be used for treatment of long and calcified lesions. On the other hand, the SFPA is subject to longitudinal stretching, external compression, torsion and flexion, which may lead to stent fractures and eventually to restenosis. While evolution in stent material and design has overcome some of these limitations, the clinical impact remains unclear.

Selection of the primary endovascular treatment for patients with SFPA disease with either PTA or stenting as the initial approach was assessed in multiple randomized trials producing conflicting results [90-98], with two meta-analyses demonstrating overall no difference in primary patency rates between the two endovascular techniques [79, 80]. A previous cost-effective analysis of a trial that randomized patients with SFPA disease to treatment with a self-expanding nitinol stent versus PTA demonstrated that the use of routine stenting increased the procedure duration, equipment costs and physician services, resulting in initial hospital costs $3,500/patient higher for patients randomized to the nitinol stent compared to PTA. The authors of that study concluded that a strategy of routine stent implantation for patients with SFPA disease is not optimal on economic grounds and that PTA with provisional stenting should be preferred [99]. Based on current evidence and lower equipment cost, recent guidelines recommend PTA as the initial preferred option for endovascular treatment of symptomatic SFPA lesions with bail-out stent placement after a suboptimal or failed result from balloon dilation [1, 7].

Stent designs have changed over the years, however, and a recent randomized trial involving 104 patients with severe claudication or critical limb ischemia showed significantly higher patency rates at 1 and 2 years for long SFPA lesions treated with primary nitinol stent implantation compared to PTA with provisional stenting (63% and 37% at 1 year; 54.3% and 30.8% at 2 years, respectively) [97, 98]. This finding supports the concept that the improvement in nitinol stent design, including improvement in radial strength, the ability to recover from being crushed and reduced foreshortening, has led to better anatomical and clinical outcomes than the older stainless-steel stents. This is in agreement with previous studies showing superior patency outcomes with the new generation nitinol stents compared to stainless-steel stents [100, 101]. Conversely, a recent randomized trial involving 244 patients with short SFPA disease using a different self-expandable nitinol stent demonstrated similar restenosis rates at 1 year with primary stenting and PTA with provisional stenting (31.7% and 38.6%, respectively).

The frequency of nitinol stent fractures in these two recent trials was only 2% and 12% at 12 months, respectively [96, 98] and in the study with the highest frequency of stent fractures the binary restenosis rate was similar in fractured and non-fractured stents [98]. However, the occurrence of stent fractures in SFPA and their association with in-stent restenosis remains an important consideration. A trial investigating the occurrence and the clinical impact of stent fractures after femoropopliteal stenting with nitinol stents suggested a considerable risk of stent fractures (24.5%), especially following long segment femoral artery stenting. Stent fractures in this study were associated with a higher in-stent restenosis and re-occlusion rate after a mean follow-up of 11 months [102].

Based on the discrepancy of these results and the improvement of stent designs over the years, more data is required to elucidate whether other factors, such as the length of the stented segment as well as the stent design and stent surface, are more likely to affect stent patency than stent fractures. Currently, there are three ongoing randomized trials in the recruitment phase comparing nitinol stents (SMART Cordis, Miami, FL) with balloon angioplasty for SFPA disease as well as one trial comparing two different types of nitinol stents (Table **1**).

#### Drug-Eluting Stents and Balloons

Initial attempts at transferring the benefits seen with drug eluting stents in the coronaries to the femoropopliteal arteries have not yet been successful. The SIROCCO II study, a randomized trial of sirolimus-coated nitinol stents compared to bare metal nitinol stents for SFPA disease, demonstrated no significant difference in patency rates. At 24 months of follow-up, the restenosis rate in the sirolimus group was 22.9% versus 21.1% in the bare metal stent group [103]. Currently, there is an ongoing large randomized trial (ZILVER PTX) in the recruitment phase using a paclitaxel-coated nitinol stent versus angioplasty in the treatment of symptomatic SFPA disease (Table **1**).

Recently, promising results were published from a randomized multicenter trial, assigning 154 patients with stenosis or occlusion of femoropopliteal artery to treatment with standard balloon catheters coated with paclitaxel, uncoated balloons with paclitaxel dissolved in the contrast medium, or uncoated balloons without paclitaxel [104]. Use of paclitaxel-coated angioplasty balloons during PTA for SFPA disease was associated with significant reductions in late lumen loss and target-lesion revascularization (target-lesion revascularization at 6 months, 37% in the control group vs. 4% in the group treated with paclitaxel-coated balloons; at 24 months, the rates increased to 52% and 15%, respectively). No significant benefit was seen with the use of a paclitaxel-containing contrast medium. A similar smaller randomized trial comparing paclitaxel-coated with plain balloon angioplasty in SFPA disease (PACCOCATH-FEM I) has completed enrollment (Table **1**).

#### Endografts

Early results from the use of Dacron-covered stent grafts were disappointing due to little patency benefit and high early and late restenosis rates, with a considerable rate of inflammatory complications, such as fever and pain [105]. However, the development of new self-expandable stent endografts, with an expanded polytetrafluoroethylene tube inside a nitinol support structure, yielded more promising results. A prospective single-arm safety and efficacy study of the Hemobahn endoprosthesis (W. L. Gore & Associates, Flagstaff, AZ) in 80 limbs with occlusive femoral-popliteal lesions demonstrated a primary patency of 90% at 6 months and 79% at 12 months [106]. A single-center small randomized trial of 28 patients compared PTA with the Hemobahn endograft, , and demonstrated a statistically significant improvement in both patency and clinical outcome with the endograft [107]. Subsequent to this report, the graft delivery system was modified and it was renamed Viabahn endoprosthesis. A prospective single-arm study of 87 limbs in 76 patients with long atherosclerotic occlusive disease of the SFPA (mean lesion length 14.2 cm, with 92% of lesions with at least 7 cm in length) treated with the Viabahn endograft demonstrated primary patency rates 76% at 1 year and 55% at 4 years [108]. Furthermore, a recent randomized prospective study of 100 patients comparing percutaneous treatment of SFPA disease with the Viabahn stent graft and surgical bypass with synthetic graft showed similar primary patency rates at 12 months in both groups (73.5% and 74.2%, respectively) [109]. The Viabahn endoprosthesis is currently approved by the US Food and Drug Administration (FDA) for use in patients with symptomatic superficial femoral arterial lesions with reference vessel diameters of 4.8 to 7.5 mm and unpublished data from the FDA approval letter randomizing 197 limbs with SFPA disease to Viabahn stent graft vs. PTA demonstrated similar technical success, primary patency and target vessel revascularization (TVR) rates at 12 months [110]. Notably, when “technical success” and “primary patency” were re-defined to be consistent with the literature, these endpoints appeared favoring the Viabahn endograft. At present, there is an ongoing randomized, prospective, multicenter trial examining the performance of the Viabahn endoprosthesis compared to bare metal nitinol stents in long SFPA lesions (>8cm) with a planned 3-year follow-up (Table **1**). The results of this trial may provide more solid evidence supporting the use of this technology.

#### Adjunctive Endovascular Therapies

There are many emerging adjunctive endovascular technologies, such as atherectomy, laser angioplasty, cutting balloon angioplasty, cryoplasty and brachytherapy, that have been investigated and their role remains controversial.

Directional atherectomy was developed with the idea to increase procedural luminal gain while avoiding barotraumas and vessel recoil. Initial results using the Simpson AtheroCath (Guidant, Temecula, CA) were disappointing suggesting that directional atherectomy might actually be worse than plain angioplasty [111]. However, in the recent years there has been a new interest in debulking devices with the development of a new atherectomy system, the SilverHawk Atherectomy Catheter (Fox Hollow Technologies, Redwood City, CA) as well as excimer laser atherectomy catheters. There are no prospective randomized trials comparing excisional atherectomy with the SilverHawk catheter to PTA or stenting. The initial long-term outcomes from a single-center non-randomized study demonstrated primary patency at 18 months of 73% for de novo lesions and much lower for native vessel restenosis (42%) or in-stent restenosis (49%) [112]. The potential role of excisional atherectomy with the SiverHawk catheter for the treatment of critical limb ischemia is to be further evaluated in an ongoing randomized trial comparing the SilverHawk atherectomy with surgical bypass (PROOF trial: Plaque Removal versus Open Bypass Surgery for Critical Limb Ischemia). New devices that permit simultaneous rotational atherectomy and aspiration of plaque material or thrombus have been recently designed and evaluated in pilot studies [113].

Excimer laser atherectomy uses flexible fiberoptic catheters to deliver intense bursts of ultraviolet energy in short pulse durations with the potential advantage to treat long occlusions and complex disease. This technology has been evaluated for long SFPA occlusions (mean length of 19.4 cm) with high immediate technical success (90.5%), but disappointing 1-year primary patency rates (65.1%). However, intensive surveillance using objective testing followed by prompt repeat intervention demonstrated 1-year secondary patency rates of 75.9% [114]. Results have been more promising for the treatment of critical limb ischemia with laser angioplasty in the Laser Angioplasty for Critical Limb Ischemia (LACI) trial, a multicenter prospective registry of 155 limbs with complex, long (>16 cm) and diffuse disease, demonstrating an excellent limb salvage rate of 93% at 6 months [115]. A major limitation of the laser atherectomy catheters has been the inability to create a channel much larger than the catheter diameter. To overcome this limitation, a new design of laser catheter has been developed, the TURBO-Booster catheter (Spectranetics, **Colorado Springs, CO**), which allows the laser to directionally ablate tissue to obtain a larger diameter. The safety and efficacy of this device is currently being evaluated in the multicenter CELLO trial (ClirPath Excimer Laser to Enlarge Lumen Openings).

Remote endarterectomy is a hybrid of minimally invasive surgery and endovascular techniques for treatment of long segment SFPA lesions with a single small groin incision. Despite the initial disappointing results [116], a recent study combining this technique with placement of an aSpire stent, an expanded polytetrafluoroethylene (ePTFE) covered nitinol stent with high radial strength, in long SFPA lesions (mean length 28.2 cm) demonstrated a primary cumulative patency rate of 60.6% at 33 months, with 25% of patients requiring secondary PTA and/or stent during follow-up [117]. At present there is an ongoing randomized control trial evaluating remote endarterectomy with placement of an aSpire stent versus bypass surgery in patients with long (>10 cm) symptomatic superficial femoral artery disease (REVAS - Remote Endarterectomy Versus Suprageniculate Femoropopliteal Bypass) with a planned 5-year follow-up.

Cutting balloon angioplasty is another evolved endovascular technology with the idea to reduce stretching and overexpansion of the vessel wall by inducing longitudinal cuts into the plaque and the inner layers of the vessel wall, with the potential of use in calcified, rigid and undilatable lesions. A small randomized study to evaluate cutting balloon angioplasty in comparison to plain balloon angioplasty for SFPA disease was terminated prematurely after the device has been recalled by the manufacturer due to potential shaft separation of the catheter [118].

Another plaque modulation technology that has been evaluated with equivocal results is cryoplasty, using an automated liquid nitrogen deployment system to temporarily freeze the intima and media wall layers during balloon dilation with the thought to induce a more benign healing process and reduce neointima formation and subsequent restenosis following angioplasty. In a multicenter prospective cohort of 102 patients with symptomatic SFPA disease (mean lesion length 4.7 cm), this technique had an immediate technical success rate of 85.3%, primary patency rate by duplex ultrasound of 70.1% at 9 months and clinical patency rate (defined as freedom from target lesion revascularization) of 75% at 3 years [119, 120]. These results appear very similar to plain angioplasty and, in the absence of comparative results as well as higher device cost, this technology is not justified for general application.

Similarly, trials of brachytherapy, using a catheter to deliver radiation to the lesion in adjunction with PTA to prevent restenosis, have yielded inconsistent results. In a randomized trial adjunctive endovascular brachytherapy (EVBT) at a dose of 12 to 14 Gy combined with PTA in SFPA long stenoses was compared with PTA alone. The seemingly beneficial short-term effects of EVBT with PTA were not sustained at 5-year follow-up, with no robust differences compared to PTA alone [121]. Likewise, the 5-year results from the prospective randomized Vienna-2 trial, which was designed to evaluate the safety and effectiveness of adjunctive EVBT compared with no further treatment after successful revascularization in patients with long-segment femoropopliteal lesions demonstrated a late “catch-up” phenomenon [122]. Although at 6 months there was a significant reduction in restenosis for the PTA plus EVBT group versus the PTA alone group (29.4% *vs*. 56.9%, respectively) [123], after 5 years the recurrence rate was similar in both groups (72.5%) [122]. A similar dose regimen from the same investigators was used to prospectively evaluate the effectiveness of EVBT in the prevention of restenosis after femoropopliteal stent implantation in high-risk patients and failed to improve 6-month patency because of a high incidence of early and late thrombotic occlusion [124]. An earlier randomized trial demonstrated that late acute thrombotic occlusion in patients receiving EVBT after stenting occurred in 27% of patients undergoing EVBT with stenting, especially after discontinuation of clopidogrel treatment, versus no events occurred in the patients with stents and without EVBT or those undergoing EVBT after simple balloon angioplasty [125]. A novel approach to deliver external-beam radiation (EBR) to de novo SFPA lesions after PTA has been developed and evaluated in a randomized controlled trial. At 1 year follow-up, a single session of external beam radiation with 14 Gy of the femoropopliteal angioplasty site significantly reduced angiographic restenosis compared to the control and the lower-dose groups [126]. At the short-term, EBR was also shown to decrease restenosis rates in patients with SFPA disease treated with stents, with a lower incidence of thrombotic events than EVBT [127]. However, the late “catch –up” phenomenon observed with EVBT necessitates a longer follow-up to determine the potential benefit of external-beam radiation.

### Surgical Revascularization

Surgical treatment of lower extremity ischemia is indicated for patients with claudication and significant functional disability or critical limb ischemia, after failure of conservative or endovascular therapy, who have a reasonable likelihood of symptomatic improvement, favorable limb arterial anatomy and low cardiovascular risk for surgical revascularization [1]. With the evolution of endovascular technology and interventional techniques as well as their equivalent efficacy, lower cost and lower peri-procedural risk, surgery has become a second-line revascularization option and is currently recommended only for TASC D lesions [7]. Furthermore, surgery should be avoided, if possible, in patients younger than 50 years old, since they have a more virulent form of atherosclerosis and subsequently a higher frequency of graft failure requiring revisions and replacement [1].

Once the decision to proceed with surgical intervention is undertaken, the type of revascularization should be elected based on different variables, such as location and severity of disease, anatomy, general medical condition, prior revascularization attempts and the desired outcome. As a general rule, in patients with combined inflow and outflow disease, inflow problems are corrected first, since improvement of the inflow may diminish the symptoms of claudication and reduce the likelihood of distal graft thrombosis from low flow [1]. In the case of SFPA disease, two major factors that can modify the result of the procedure are the type of the conduit and the site of the distal anastomosis. The superior rates of immediate and long-term patency rates favor autogenous vein grafts as opposed to prosthetic conduits for both above- or below-the-knee bypasses [128-130]. The 5-year patency rates of femoropopliteal bypass grafts are reported as 80% for vein grafts, 75% for above-the-knee synthetic grafts and 65% for below-the-knee synthetic grafts [131]. Patients undergoing surgical bypass for lower extremity ischemia should be entered into a clinical surveillance program that consists of interval history and vascular exam as well as measurement of resting and, if possible, post-exercise ABIs and duplex imaging of the entire length of the graft with measurements of peak systolic velocities and calculation of the velocity ratios across all lesions in the immediate postoperative period and at regular intervals (usually every 6 months) for at least 2 years [1, 7]. Major amputation in patients with critical or acute limb ischemia should be reserved only when the limb is unsalvageable, i.e., when there is overwhelming infection that threatens the patient’s life, extensive necrosis or refractory ischemic rest pain [1,  7].

## CONCLUSION

As the population ages, it is anticipated that the prevalence of peripheral vascular disease will increase. Within the past decade there has been an unprecedented evolution of the endovascular technologies and vital improvements are expected in the next decade. Percutaneous procedures will continue to replace open surgery. The chief challenge in the management of peripheral arterial disease would be retooling of the health system to focus on identifying patients with PAD and taking the enormous opportunity and responsibility to refine and aggressively manage the atherosclerotic risk factors in these patients. An additional challenge will be to reorganize clinical and basic research to ensure that investigators are trained to design relevant studies and undertake the difficult but necessary task of collecting more definitive data to support therapeutic measures, ensure appropriateness of care and promote development of newer strategies to prevent and treat PAD.

## Figures and Tables

**Fig. (1):**
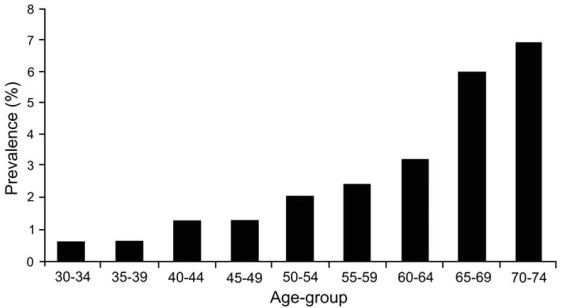
Weighted mean prevalence of intermittent claudication (symptomatic PAD) in large population-based studies. Reproduced with permission from [7].

**Fig. (2):**
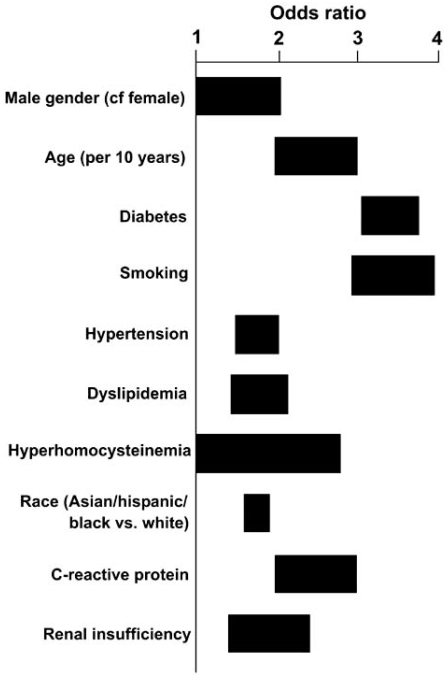
Approximate range of odds ratios for risk factors for symptomatic peripheral arterial disease. Reproduced with permission from [7].

**Fig. (3):**
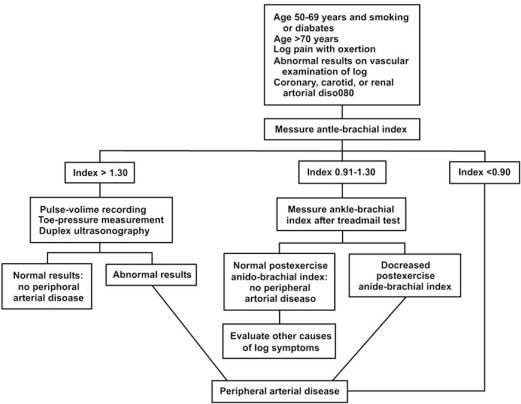
Algorithm for diagnosis of peripheral arterial disease. TBI – toe brachial index; VWF – velocity wave form; PVR – pulse volume recording. Reproduced with permission from Hiatt WR. N Engl J Med 2001; 344: 1608-21.

**Fig. (6):**
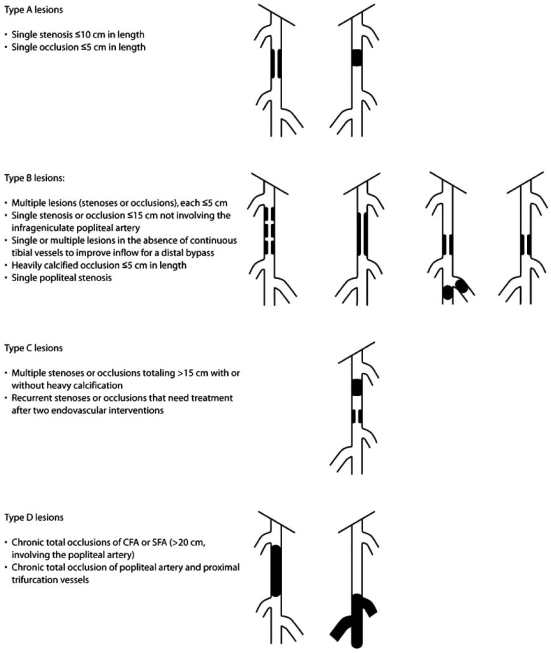
TASC classification of femoral popliteal lesions. CFA – common femoral artery; SFA – superficial femoral artery. Reproduced with permission from [7].

**Table 1:** Ongoing Randomized Controlled Trials of Balloon Angioplasty and/or Stents in Symptomatic SFA Disease. There are also five Ongoing Non-Randomized Trials Evaluating Safety and Efficacy of Different Types of Stents (NCT ID#00180505, N=120; NCT ID#00475566, N=100; NCT ID#00530712, N=287; NCT ID#00496041, N=200; NCT ID#00542646, N=45). Data from http://www.ClinicalTrials.gov

Study Name	Super UK	Sit-UP	DURAVEST	ZILVER PTX	GORE VIABAHN	SIROCCO SFA	Super SL	PACCOCATH-FEM I
Status	Recruiting	Recruiting	Recruiting	Recruiting	Recruiting	Active, Not recruiting	Active, Not recruiting	Active, Not recruiting
Phase	IV	IV	IV	I	IV	II	IV	I-II
NCT ID	00232843	00309595	00289055	00120406	00228384	00232869	00235131	00472472
Treatment arm I	Cordis SMART nitinol stent	Cordis SMART nitinol stent	Cordis SMART nitinol stent	ZILVER PTX Drug eluting stent	VIABAHN endoprosthesis	Sirolimus coated Cordis SMART nitinol stent	Cordis SMART nitinol stent	Paclitaxel-coated balloon angioplasty
Treatment arm II	Balloon Angioplasty	Balloon Angioplasty	Balloon Angioplasty	Balloon Angioplasty	Bare nitinol stent	SMART bare metal stent	Bard Luminexx stent	Plain Balloon Angioplasty
Type of study	Randomized 1:1, controlled, prospective, multicenter	Randomized 1:1, controlled, prospective, multicenter	Randomized 1:1, controlled, prospective, multicenter	Randomized 1:1, controlled, prospective, multicenter	Randomized 1:1, controlled, prospective, multicenter	Randomized 1:1, controlled, double-blinded, prospective, multicenter	Randomized 1:1, controlled, prospective, multicenter	Randomized 1:1, controlled, double-blinded, prospective, multicenter
Size (N)	150	120	120	480	150	90	200	79
Inclusion	Symptomatic SFA disease (5-14.5 cm)	Symptomatic SFA disease (5-22 cm)	Symptomatic SFA disease (5-14.5 cm)	Symptomatic SFA disease (<7 cm in phase I and <14 cm in phase II)	Symptomatic SFA disease (>8cm)	Symptomatic SFA disease	Symptomatic SFA disease (5-22 cm)	Symptomatic SFA disease
Primary endpoint	Primary patency by duplex ultrasound at 1 year	Primary patency by duplex ultrasound at 1 year	Primary patency by duplex ultrasound at 1 year	Primary patency by duplex ultrasound at 1 year	Primary patency at 3 years	In-stent stenosis via quantitative angiography at 6 months	Primary patency by duplex ultrasound at 1 year	Angiographic lumen loss at 6 months
Start date	3/2005	12/2005	11/2005	3/2005	9/2005	2/2001	5/2005	4/2004
Completion date	8/2008	4/2008	12/2008	N/A	12/2010	12/2008	3/2008	6/2007

NCT ID= ClinicalTrials.gov Identifier; SFA=superficial femoral artery; N/A= not available.
